# Identifying Targets and Drugs for Rheumatoid Arthritis Stratified Therapy Using Mendelian Randomization and a Pretraining Model

**DOI:** 10.3390/ijms26125686

**Published:** 2025-06-13

**Authors:** Yuqing Yan, You Wu, Yixuan Sun, Tian Wu, Haotian Zhu, Feiyang Xue, Zhanhui Yu, Shichao Liu, Xiaohui Niu

**Affiliations:** Hubei Key Laboratory of Agricultural Bioinformatics, College of Informatics, Huazhong Agricultural University, Wuhan 430074, China; yanyq@webmail.hzau.edu.cn (Y.Y.); wu.you@webmail.hzau.edu.cn (Y.W.); S_yix@webmail.hzau.edu.cn (Y.S.); wut@webmail.hzau.edu.cn (T.W.); 2020317110039@webmail.hzau.edu.cn (H.Z.); xuefy@webmail.hzau.edu.cn (F.X.); yuzhanhui@webmail.hzau.edu.cn (Z.Y.)

**Keywords:** rheumatoid arthritis subtypes, cytokines, pretraining model, drug prediction

## Abstract

The prevalence of rheumatoid arthritis (RA) subtypes, including seropositive and seronegative, is influenced by lifestyle factors and exhibits high heterogeneity, resulting in reduced drug efficacy. This study aims to identify cytokines mediating the effects of different lifestyles on RA subtypes and to discover new drugs for personalized treatment. Mendelian randomization revealed that three cytokines (MIP1b, SCGFb, and TRAIL) partially mediated the effects of different lifestyles on RA overall or its subtypes. The pretrained model, i.e., DrugBAN, predicted the probability of 723,000 small molecule drugs binding to these three targets. In molecules with high binding rates, we calculated the structural similarity between known drugs for RA and other drugs to screen for new drugs, followed by molecular docking and molecular dynamics simulations for validation. The results indicate that these targets had promising binding affinity with known drugs and other drugs with high similarity. Our findings may guide therapeutic approaches for heterogeneous RA patients with specific lifestyle habits.

## 1. Introduction

Rheumatoid arthritis (RA) is a prevalent chronic inflammatory disease characterized by chronic synovial joint inflammation, leading to musculoskeletal deficits and an increased risk of other diseases, such as pulmonary involvement and cardiovascular disease, thereby increasing patient mortality risk [1]. Based on the presence of several autoantibodies, such as rheumatoid factor and anti-citrullinated protein antibodies, RA can be classified into seropositive and seronegative types [2]. Seropositive RA is associated with worse outcomes, including more serious joint injury, more severe bone loss, and higher mortality [3]. While the course of seronegative RA, characterized by greater heterogeneity, is relatively mild, it can still lead to joint destruction and even disability [4]. Patients with different subtypes typically have distinct etiologies. HLA-DRB1 alleles are the strongest genetic loci for seropositive RA [5], whereas HLA-DR3 alleles contribute more to seronegative RA [6]. Furthermore, genetic factors account for approximately 50% of seropositive RA risk but only 20% of seronegative disease risk, with environmental factors contributing more to the latter [7].

RA pathogenesis involves a complex interplay between cells (such as T cells, B cells, macrophages, and synovial fibroblasts) and a network of pro-inflammatory cytokines [8]. Cytokines such as tumor necrosis factor (TNF)-α and interleukin-6 (IL6) play a central role in the disease process. TNF-α induces the expression of the receptor activator of nuclear factor κB ligand (RANKL) and synergistically promotes osteoclast differentiation, leading to bone erosion [9]. IL6 promotes the differentiation of Th17 cells, a subset of T cells, and stimulates RANKL expression, thereby inducing osteoclast formation and contributing to joint damage [10]. Combined stimulation with IL-6 and TNF-α can also induce macrophage differentiation into osteoclasts via a non-RANKL-dependent pathway [10]. However, compared with seropositive patients, seronegative individuals exhibit increased numbers of M1-like macrophages and higher levels of TNF-α expression [11].

Research on the pathology of RA led to the development of therapeutic drugs. The primary strategy for RA involves disease-modifying drugs, primarily methotrexate [12]. However, the therapeutic effect of methotrexate in seronegative patients is unsatisfactory [13], they respond better to anti-TNF [14]. Additionally, 77.5% of patients receiving methotrexate treatment experience at least one adverse event, and lifestyle may also play a role [12].

Smoking, a recognized risk factor, increases the risk of RA, especially seropositive subtypes, and exacerbates disease progression [15]. Additionally, other lifestyle factors, including diet, coffee, alcohol consumption, and body mass index (BMI), can also lead to different subtypes of RA [16,17,18,19]. For example, research by Junxiang Wang et al. shows that coffee consumption increases the risk of seronegative rather than seropositive RA [20]. Given the documented roles of certain cytokines in RA [8], and considering that lifestyle factors can modulate cytokine levels [21,22,23,24,25], incorporating cytokines as mediators in analyses may elucidate the biological mechanisms linking lifestyles to RA and its subtypes. Drugs targeting these proteins could be used for personalized therapy.

To better understand the causal relationships between lifestyle factors and RA subtypes, and to explore the potential mediating role of cytokines, we conducted two-sample Mendelian randomization (MR) analyses based on genome-wide association study (GWAS) summary statistics. This method uses genetic variants as instrumental variables for exposures, thereby minimizing confounding and reverse causality [26]. Compared to conventional observational studies, MR enables more robust causal inference. Additionally, to translate the findings into therapeutic implications, we employed the DrugBAN model [27] to predict the binding probabilities between small molecule drugs and cytokines with potential mediating roles. DrugBAN takes molecular graphs and protein sequences as inputs and learns joint representations through a bilinear attention mechanism. In benchmark evaluations, DrugBAN outperformed both traditional machine learning and other deep learning models, particularly in predicting drug–target pairs across different data distributions. These advantages make it a suitable tool for identifying candidate drugs targeting cytokines related to RA. Our findings, based on the integration of MR and DrugBAN, could identify novel therapeutic targets and drugs for patients with specific RA subtypes associated with particular lifestyle habits, potentially leading to more personalized and effective treatment strategies.

## 2. Results

**Figure 1:**
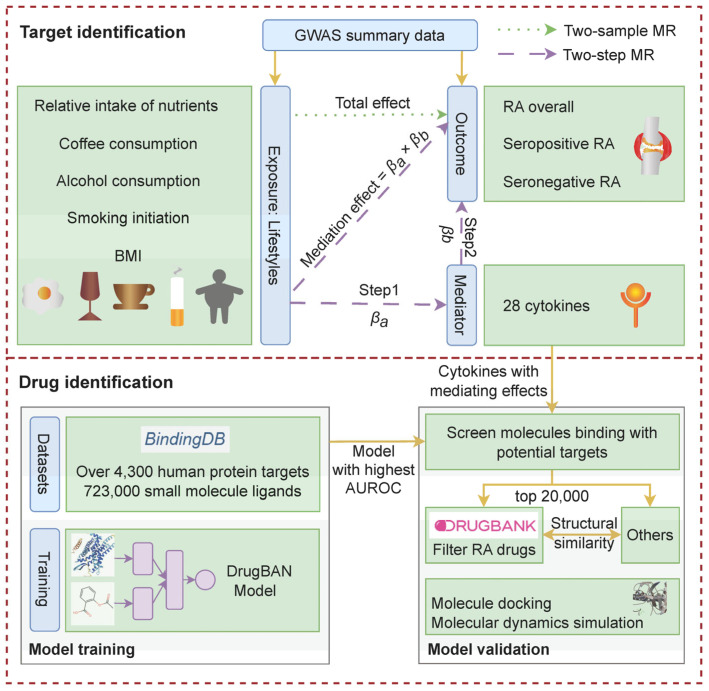
Flow chart of the study designed in this MR study. AUROC, area under the receiver operating characteristic.

To investigate the cytokines that mediate the impact of lifestyle factors on RA and its subtypes, and to identify new targeted drugs, we collected available large-scale GWAS summary data of lifestyles, cytokines, and RA (Table 1) and designed the following pipeline (Figure 1).

### 2.1. Total Effects of Lifestyles on RA and Its Subtypes

To evaluate the total effects of lifestyles on RA and its subtypes, we conducted two-sample MR analyses using genetic variants as instrumental variables. In total, 9, 4, 7, 6, 14, 83, 334, and 752 SNPs were selected for relative intake of four nutrients (carbohydrate, fat, protein, and sugar), coffee consumption, alcohol consumption, smoking initiation, and BMI, respectively (Tables S1–S8). Four MR methods were applied, including inverse variance weight (IVW), weighted-median (WM), MR-Egger, and MR pleiotropy residual sum and outlier (MR PRESSO). The results of the IVW method are considered the primary findings.

**Figure 2:**
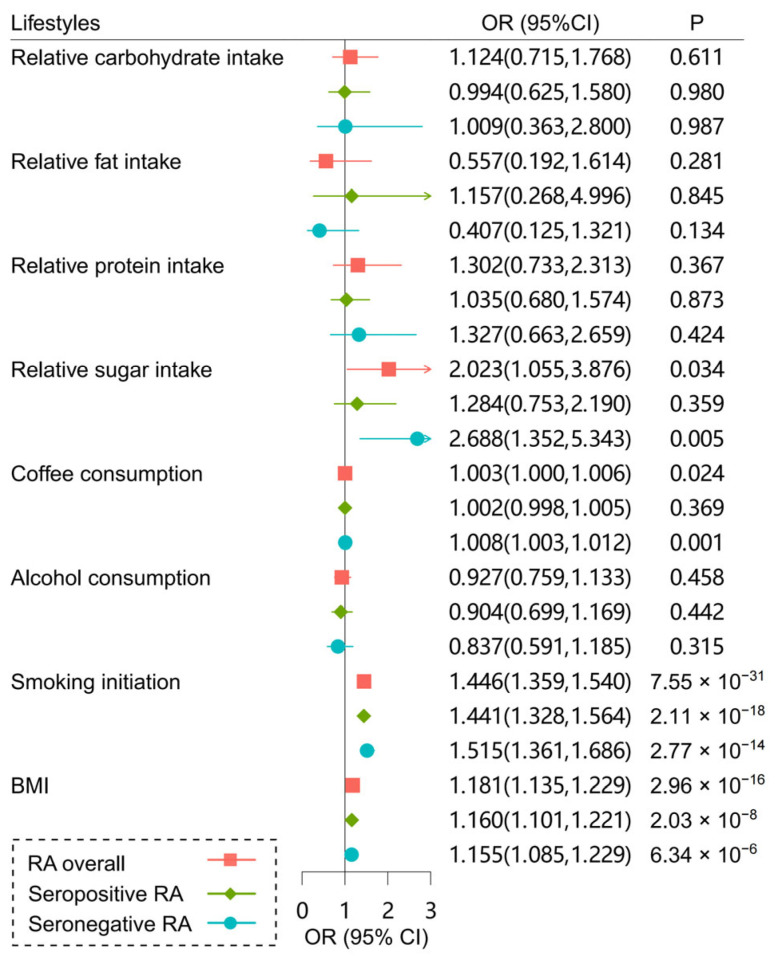
Forest plot of the causal associations of lifestyles with RA and its subtypes. *p* < 0.05 was considered as significant evidence of associations.

As shown in Figure 2, the risks of RA overall and its subtypes were positively associated with smoking initiation and BMI, consistent with previous observational studies [34,35]. We also observed subtype-specific effects for several lifestyle factors. Increased relative sugar intake was associated with a higher risk of seronegative RA (odds ratio [OR] = 2.688, *p* = 0.005) but not seropositive RA (OR = 1.284, *p* = 0.359). Higher coffee consumption was causally associated with a higher risk of seronegative RA (OR = 1.008, *p* = 0.001) but did not significantly affect seropositive RA (OR = 1.002, *p* = 0.369). However, no significant associations were found between RA risk and relative intake of macronutrients (carbohydrate, fat, and protein) or alcohol consumption.

Most results of sensitivity analyses (WM, MR-Egger, and MR PRESSO) were consistent with the IVW method (Figures S1–S3). Cochran’s Q statistics indicated no significant heterogeneity (*p* > 0.05, Table S9). MR-Egger showed pleiotropy in the effects of BMI on RA overall and its subtypes (*p* < 0.05). However, the MR PRESSO method detected no outliers and supported these associations (Table S9).

### 2.2. Cytokines as Potential Targets

Cytokines are critical in the development of RA and its subtypes [8,9,10], and lifestyles can modulate cytokine levels [22,23,24]. Therefore, we conducted two-step MR analyses to elucidate the mediating pathways through which lifestyle factors influence RA and its subtypes via 28 cytokines. We concentrated on pathways from lifestyles to diseases that demonstrated significant associations (*p* < 0.05) in the two-sample MR analyses (Figure 2). Cytokines involved in these pathways might be potential therapeutic targets for RA patients with specific lifestyle exposures.

**Figure 3:**
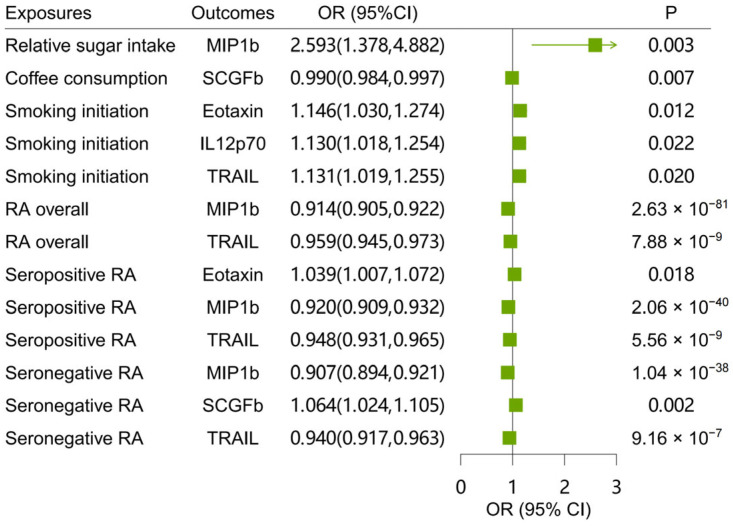
Significant associations in the two-step MR analyses. The results with *p* < 0.05 are considered significant.

In the first step, we used genetic variants for lifestyles to assess their causal effects on cytokines. We identified five cytokines significantly associated with lifestyles (Figure 3 and Figures S4–S7). In the second step, we evaluated the causal effects of these five cytokines on the risk of RA overall and its subtypes. There were 51, 19, 297, 36, and 65 SNPs USED as instrumental variables for eotaxin, IL12p70, macrophage inflammatory protein-1b (MIP1b), stem cell growth factor-beta (SCGFb), and TNF-related apoptosis inducing ligand (TRAIL), respectively (Table S10). Our findings indicate that decreased levels of MIP1b and TRAIL led to a higher risk of RA overall and its subtypes. Eotaxin and SCGFb levels were positively associated with the risk of seropositive and seronegative RA, respectively (Figure 3 and Figures S8–S10). Sensitivity analysis results show no heterogeneity and horizontal pleiotropy within the MR analyses (Tables S11 and S12).

Finally, we estimated the indirect effects of lifestyles on RA and its subtypes via these cytokines. By integrating the results from two-sample MR analyses and two-step MR analyses, we identified that seven mediation pathways (Table 2) and six pathways were statistically significant (*p* < 0.05), in total involving three cytokines (MIP1b, TRAIL, and SCGFb). We observed significant indirect effects of relative sugar intake on RA overall and seronegative RA through MIP1b, with mediation proportions of 12.19% (*p* = 0.004) and 9.36% (*p* = 0.004), respectively. TRAIL partially mediated the effects of smoking initiation on RA overall, seropositive RA, and seronegative RA, with mediation effects of −0.005 (*p* = 0.033), −0.007 (*p* = 0.033), and −0.008 (*p* = 0.039), and mediated proportions of 1.41%, 1.80%, and 1.84% in the total effect, respectively. The mediation effect of coffee consumption on seronegative RA through SCGFb was −0.001 (*p* = 0.047), with a mediated proportion of 7.84%.

Among the three significant mediators mentioned above, TRAIL has been proposed as a potential therapeutic target for RA [36]. The association between MIP1b and RA remains debated in the literature [37,38,39], while research on SCGFb is relatively sparse.

### 2.3. Candidate Drugs Prediction

To identify small molecules targeting cytokines with mediating roles (MIP1b, TRAIL, and SCGFb), we trained the DrugBAN model using a comprehensive dataset from BindingDB, which included over 4300 human protein targets and 723,000 small molecule ligands, and identified 1.45 million valid binding data pairs.

Then, we used the model with the highest area under the receiver operating characteristic (AUROC = 0.980) score to predict the binding probabilities of the three cytokines mentioned above with small molecules. Among these, eleven small molecules for MIP1b, three for TRAIL, and eight for SCGFb were found to be validated for RA treatment in the DrugBank database (Table 3).

**Figure 4:**
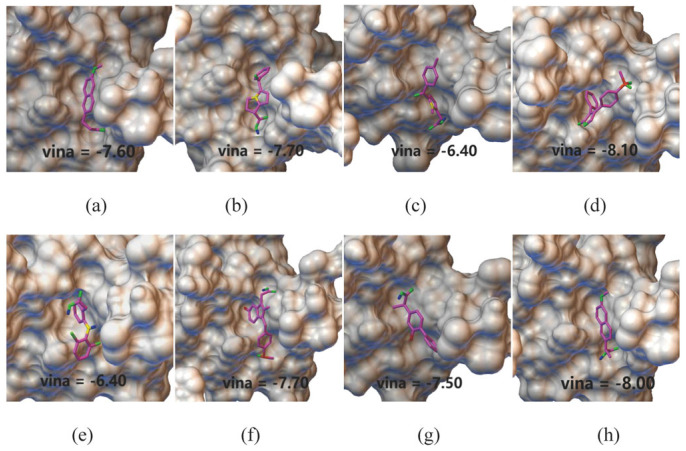
The simulated docking conformations of SCGFb with the identified drugs. (**a**) Nabumetone; (**b**) ketorolac; (**c**) tolmetin; (**d**) rofecoxib; (**e**) diclofenac; (**f**) sulindac; (**g**) flurbiprofen; and (**h**) naproxen.

To further validate the model prediction results, we used AutoDock Vina to analyze the binding sites and interactions between the three cytokines (MIP1b, TRAIL, and SCGFb) and RA treatment drugs. We selected the conformation with the lowest docking score as the best conformation. For instance, the lowest binding scores for the eight small molecules docked with SCGFb ranged from −6.40 to −8.10 kcal/mol (Table 3), indicating the good binding affinity of these small molecules. Notably, the lowest docking score for SCGFb with Rofecoxib was −8.10 kcal/mol, suggesting its potential as a strong inhibitor (Figure 4). In addition to Rofecoxib, Figure 4 also presents the docking scores of SCGFb with the other seven drugs identified from our search in the DrugBank database for approved RA treatments. Similarly, the docking simulations for MIP1b and TRAIL showed promising binding affinities (MIP1b: −6.0 to 7.6 kcal/mol and TRAIL: −7.7 to 8.2 kcal/mol, respectively), and supported their potential therapeutic relevance in RA treatment. These docking scores confirmed the binding potential of these small molecules, suggesting that these three protein targets are likely involved in RA treatment.

**Figure 5:**
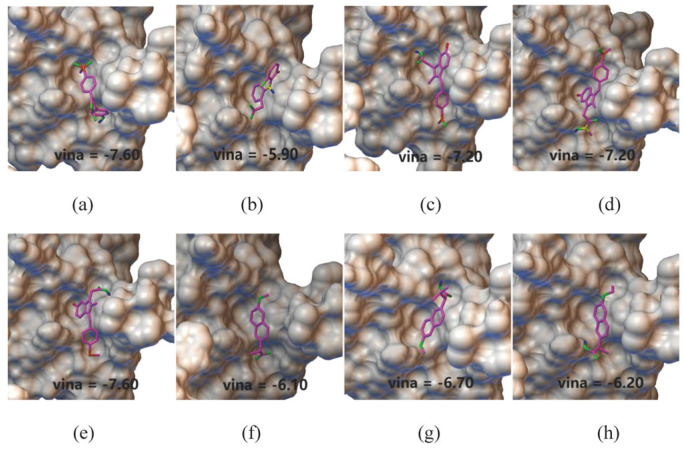
Docking conformations of the top eight molecules ranked by similarity to approved drugs for SCGFb. PubChem CID: (**a**) 10359269; (**b**) 3032; (**c**) 1548885; (**d**) 44305866; (**e**) 5352624; (**f**) 6926388; (**g**) 116964097; and (**h**) 20334995.

For other small molecules predicted by DrugBAN, those with high binding probabilities to MIP1b, TRAIL, and SCGFb and with structural similarities to drugs already approved for RA treatment were considered potential candidates for further investigation. We used the Tanimoto similarity coefficient [40], a widely used metric in the field of drug discovery, to evaluate the structural similarity between molecules. Given that TRAIL is a known drug target for RA [36] and SCGFb has potential as a novel target, we calculated the similarity between known approved drugs and the top 20,000 ranked molecules for these two targets. For SCGFb, we conducted molecular docking simulations on the eight molecules with the highest similarity, as shown in the Figure 5, which demonstrated strong binding potential, with scores ranging from −5.9 to −7.6 kcal/mol. Similarly, for TRAIL, the top eight molecules had binding scores ranging from −6.7 to −8.3 kcal/mol, indicating strong binding potential. This approach suggests that screening for small molecules with similar functions is feasible and may yield new candidate drugs for personalized RA treatment.

To further validate their binding probabilities, molecular dynamics (MD) simulations were performed for each of the eight candidate molecules with the highest structural similarities in the SCGFb complexes, followed by the MM-GBSA energy calculations. Two ligands (PubChem CID: 6926388 and 20334995) dissociated from the binding site of SCGFb (Figure S11); thus, their binding energies were not computed. Table 4 shows qualitative agreement among the predicted binding probabilities, docking scores, and calculated binding energies. The ligand with the highest binding probability (PubChem CID: 44305866) had the second strongest binding strength indicated by both the docking score and MM-GBSA energy. In addition, the two ligands dissociated from the SCGFb binding site (PubChem CID: 6926388 and 20334995) had the second and third weakest binding strengths according to the docking scores, one of which (PubChem CID: 20334995) also had the lowest binding probability (Table 4). Although the linear correlation among the binding probabilities, docking scores, and binding energies was not obvious, their qualitative agreement from the MD simulations and MM-GBSA energy calculations provided additional support for the predictions from the DrugBAN model and their subsequent validation through molecular docking.

## 3. Discussion

This study identified three cytokines (MIP1b, TRAIL, and SCGFb) as mediators of lifestyle-induced RA and its subtypes through MR analyses. To discover personalized drugs targeting these targets, we trained the DrugBAN model to predict the binding probabilities of small molecules to these targets. The reliability of our predictions was supported by summary data from the DrugBank database and molecular docking scores. Small molecules with high predictive probabilities and structural similarities to known RA drugs may represent promising new drugs.

In terms of cytokine-mediated effects, our two-step MR analyses provide genetic evidence supporting a causal pathway from smoking to TRAIL expression and further to the risk of RA and its subtypes, extending previous findings. TRAIL, a member of the TNF superfamily, is involved in immune and inflammatory responses [36]. Microarray analysis has shown that smoking is associated with increased TRAIL levels, triggering an inflammatory response in adipocytes [41]. RA synovium is characterized by the presence of aggressively activated synovial fibroblasts that destroy cartilage and bone. Research shows that patient synovial cells can express TRAIL receptors, thereby inducing apoptosis [42]. However, TRAILs may trigger the formation of a secondary signal complex, activating nuclear factor-κB and mitogen-activated protein kinases, leading to a proinflammatory reaction [42]. In vitro experiments further support this, demonstrating that TRAIL induces rapid apoptosis in up to 30% of synovial cells within the initial 24 h, followed by increased synovial cell proliferation activity, potentially rendering them more resistant to TRAIL-induced apoptosis [43]. Moreover, our study highlights subtype-specific mediation effects. We found that the mediation proportion of TRAIL was smaller in seropositive RA than in seronegative RA. This may be related to differences in T cell subsets expressing TRAIL, as seronegative patients tend to exhibit lower levels of T cells [44,45]. The stronger protective mediation effect of TRAIL in seronegative RA suggests that TRAIL signaling may be of therapeutic relevance in this subgroup, especially among smokers. Nevertheless, this hypothesis warrants experimental validation.

Additionally, SCGFb played a mediating role in the pathway from coffee consumption to seronegative RA. However, to the best of our knowledge, there is limited data on the pathophysiological role of SCGFb. SCGFb is a shorter form of SCGF encoded by the gene CLEC11A, which supports the growth of hematopoietic progenitor cells [46]. SCGF is highly expressed in the endothelium of synovial tissue in RA patients and may participate in synovial infiltration by regulating endothelial cell function [47]. Some other activating C-type lectins, such as CLEC5A, demonstrated pro-inflammatory activity, and the loss or inhibition of related receptors has shown a protective effect in a murine model of RA [48].

MIP1b, also known as CCL4, a CC chemokine, significantly mediated the effects of relative sugar intake on RA in our study, especially in the seronegative rather than seropositive subtype. While previous studies established a causal relationship between MIP1b and RA [37,38], our findings highlight MIP1b as a mediator linking sugar intake to RA, a pathway that remains underexplored in RA research. However, the negative association between MIP1b and RA in this study contradicts previous reports of higher MIP1b levels in RA patients [39]. Further investigation is necessary to elucidate how MIP1b influences the development of RA.

Although targeted drugs have been developed for some cytokines, many problems still remain. TNF and IL6 inhibitors can increase the incidence of some malignant tumors [48] and the risk of Mycobacterium tuberculosis reactivation [49]. Anakinra, a recombinant IL-1R antagonist, is safer than anti-TNF therapies, but has lower efficacy in most patients [50]. Therefore, it is necessary to develop new drugs to achieve better safety and higher response rates. Yu Jeong Kim et al. developed ionic complex systems based on hyaluronic acid and polyethylene glycol-derivatized TRAIL, which showed excellent therapeutic effects for RA treatment in an arthritis mouse model [51,52]. Moreover, in patients with confirmed RA, TRAIL concentrations increased more in those with heart failure compared to those without [53]. This finding suggests that treatment aimed at reducing the inflammatory burden caused by TRAIL in RA patients may prevent heart failure. Although there is no strategy aimed at SCGFb for RA treatment, the relevant drugs are still worthy of consideration due to the key role of SCGFb in osteoblast differentiation [54].

Some small molecules predicted by DrugBAN to bind these targets have been validated in the DrugBank. By evaluating structural similarities between small molecules with high binding probabilities and known drugs using the Tanimoto similarity coefficient [40], we suggest that screening small molecules with similar functions could identify new candidate drugs for RA treatment.

## 4. Materials and Methods

### 4.1. MR Data Sources

We collected GWAS summary statistics for eight lifestyle factors: relative intake of carbohydrate, fat, protein, and sugar [28], coffee consumption [29], alcohol consumption [30], smoking initiation [30], and BMI [31] (Table 1). The summary data for RA and its subtypes were obtained from a recent GWAS meta-analysis, comprising 31,313 RA cases (68% seropositive) and approximately one million controls from European populations [32]. The samples were subclassed as seropositive and seronegative RA based on rheumatoid factor and anti-cyclic citrullinated peptide measurements [32]. We utilized 28 available cytokines as potential mediators, selected from a GWAS involving 8293 participants [33].

### 4.2. Two-Sample MR Analysis and Sensitivity Analysis

We first selected single nucleotide polymorphisms (SNPs) achieving genome-wide significance (*p* < 5 × 10^−8^) as instrumental variables to represent genetic susceptibility to exposure [55]. Subsequently, SNPs were clumped to exclude those with linkage disequilibrium (*r*^2^ > 0.01 and clump window < 10 kb) [55]. Additionally, SNPs directly associated (*p* < 5 × 10^−8^) with the outcome were excluded [56] to ensure that instrumental variables influenced the outcome solely through exposure. SNPs were then harmonized to ensure allele correspondence between exposure and the outcome. We also employed the RadialMR (version 1.0) package [57] to remove outlier pleiotropic SNPs (*p* < 0.05). SNPs with an F statistic (*β*^2^/*SE*^2^ < 10) were excluded to avoid weak instrument bias [58]. The remaining SNPs were utilized for MR analyses.

We employed two-sample MR analyses to evaluate the total effects of lifestyles on RA and its subtypes, using the IVW model as the primary method [59]. For associations with *p* < 0.05, we estimated the causal effects of these lifestyles on cytokines. Subsequently, cytokines associated with lifestyles (*p* < 0.05) were used to evaluate their causal effects on RA and its subtypes. Finally, we applied the “product of coefficients” method [60] to assess the indirect effects of lifestyles on RA and its subtypes through cytokines. The Aroian test was used to calculate standard errors for the indirect effects using the online tool (http://www.quantpsy.org/sobel/sobel.htm, accessed on 25 November 2023). Cytokines with *p* < 0.05 are considered potential therapeutic targets.

Three additional methods, including WM [61], MR-Egger [62], and MR PRESSO (MRPRESSO package, version 1.0) [63], were also employed to enhance the robustness of our MR analyses. Cochran’s Q statistic was used in the IVW and MR-Egger methods to evaluate heterogeneity [64].

### 4.3. Candidate Drug Prediction Using Deep Learning Models

#### 4.3.1. Principle of DrugBAN

To identify small molecule drugs that interact with potential targets, we trained the DrugBAN model and predicted the probability of small molecules binding to these targets.

DrugBAN, based on a bilinear attention network, is designed to capture local interactions between drugs and proteins. In DrugBAN, protein sequences are encoded using a three-layer one-dimensional convolutional neural network. Drug compounds are represented as two-dimensional molecular graphs derived from SMILES strings, with each atom node initialized based on its chemical properties to form a node feature matrix. A three-layer graph convolutional network is employed to learn graph representations by aggregating neighborhood information. The BAN module captures pairwise local interactions between drug and protein features using third-layer hidden representations to generate a joint representation through bilinear pooling. This is followed by sum pooling to create a compact feature map, which is fed into a fully connected layer, with the predicted probability obtained via the sigmoid function.

#### 4.3.2. Datasets and Model Training

To train the DrugBAN model, we sourced a comprehensive dataset of small molecule protein-binding interactions from BindingDB (https://www.bindingdb.org/rwd/bind/index.jsp, accessed on 16 May 2024). This dataset included over 4300 human protein targets and 723,000 small molecule ligands. We followed the same training procedures described in the original study [27] and selected the optimal model based on the highest AUROC score achieved on the validation set for subsequent screening.

#### 4.3.3. Model Validation

The trained DrugBAN model was used to screen small molecules for their potential to bind with protein targets. Molecules were ranked according to their predicted binding probabilities. We selected the top 20,000 small molecules as candidate drugs based on their rankings and searched the DrugBank database (https://go.drugbank.com/) for any that have been approved for RA treatment. We then used the Tanimoto similarity coefficient [40] to evaluate the structural similarity between candidate drugs and known RA drugs to identify new potential drugs.

To further validate these results, we performed molecular docking simulations using AutoDock Vina v1.1.2 [65]. Potential targets identified through MR analyses were docked with their corresponding small molecules for RA treatment. Protein structure data were downloaded from the PDB (https://www.rcsb.org/), and small molecules were retrieved from DrugBank in sdf format. All files were converted to pdbqt format before docking. Each protein–ligand pair generated eight conformations along with their corresponding docking scores. We selected the conformation with the lowest docking score as the best conformation, indicating the strongest binding affinity.

The stability of the top 8 SCGFb-binding candidates in the binding site was further validated by MD simulations. The top-ranked docked pose in each docking result was extracted and used to build the molecular models for SCGFb-binding complexes by the tLEaP module with valence parameters from the GAFF force field in AMBER18 [66]. The atomic partial charges for the 8 SCGFb-binding candidates were computed by the AM1-bcc method [67]. Each complex structure was solvated with TIP3P water using an 8 Å buffer in a truncated octahedral box using the tLEaP module of the AMBER18 software package. Sodium counter ions were added to neutralize the solvated system. The MD simulation protocol for each system was adopted from Roe’s study [68]. The restraint weight in the relaxation steps was increased to 10 kcal/mol·Å^2^ in the present study. The production MD simulations for each system were performed with the GPU implementation [69] of the PMEMD.CUDA module for 100 ns and trajectory frames were collected at every 1 ps. In all MD simulations, covalent bonds involving hydrogen atoms were constrained using the SHAKE algorithm [70], allowing a simulation time step of 2 fs. A nonbonded cutoff of 8 Å was applied to van der Waals interactions, with long-range electrostatics treated with the particle mesh Ewald approximation. The interaction energies in each SCGFb-binding complex were computed with the molecular mechanics-generalized born solvent-accessible surface area (MM-GBSA) methodology under the single trajectory methodology [71,72]. The MMPBSA.py.MPI module and 10,000 evenly extracted snapshots from each MD simulation were applied for each MM-GBSA calculation, in which the GB_1_^OBC^ model [73] (igb = 2) and internal dielectric constant (ε_int_) of 4.0 were used [74].

## 5. Conclusions

The effects of lifestyles on RA, including subtypes, were partially mediated by MIP1b, TRAIL, and SCGFb, informing interventions for RA patients with specific lifestyle habits. Drugs predicted for these targets have the potential to be used for personalized therapy.

## Figures and Tables

**Table 1:** Summary information of data in this study.

Type	Trait	Sample Size (Cases)	Unit	Citation
Exposure	Relative carbohydrate intake	268,922	% of total energy intake	[28]
	Relative fat intake	268,922	% of total energy intake	[28]
	Relative protein intake	268,922	% of total energy intake	[28]
	Relative sugar intake	235,391	% of total energy intake	[28]
	Coffee consumption	375,833	50% change	[29]
	Alcohol consumption	941,280	1-SD increase in log-transformed alcoholic drinks per week	[30]
	Smoking initiation	1,232,091	Ever smoked regularly compared with never smoked	[30]
	BMI	681,275	kg/m^2^	[31]
Outcome	RA overall	1,026,690 (31,313)	Odds ratio	[32]
	Seropositive RA	1,009,623 (18,019)	Odds ratio	[32]
	Seronegative RA	1,023,986 (8515)	Odds ratio	[32]
Mediator	28 cytokines	840–8293	SD	[33]

These studies were all based on European ancestry. RA: rheumatoid arthritis; SD: standard deviation.

**Table 2:** Mediating effects of lifestyles on RA and its subtypes via cytokines.

Outcome	Exposure	Mediator	Total Effect	Mediation Effect		Mediated Proportion
			*β* (95% CI)	*β* (95% CI)	*p*	
RA overall	Relative sugar intake	MIP1b	0.704 (0.054, 1.355)	−0.086 (−0.144, −0.028)	0.004	12.19%
RA overall	Smoking initiation	TRAIL	0.369 (0.306, 0.432)	−0.005 (−0.010, 0.000)	0.033	1.41%
Seropositive RA	Smoking initiation	Eotaxin	0.366 (0.284, 0.448)	0.005 (−0.001, 0.011)	0.097	1.42%
Seropositive RA	Smoking initiation	TRAIL	0.366 (0.284, 0.448)	−0.007 (−0.013, −0.001)	0.033	1.80%
Seronegative RA	Relative sugar intake	MIP1b	0.989 (0.302, 1.676)	−0.093 (−0.156, −0.029)	0.004	9.36%
Seronegative RA	Coffee consumption	SCGFb	0.008 (0.003, 0.012)	−0.001 (−0.001, 0.000)	0.047	7.84%
Seronegative RA	Smoking initiation	TRAIL	0.415 (0.308, 0.522)	−0.008 (−0.015, 0.000)	0.039	1.84%

**Table 3:** Drugs with the potential to bind to SCGFb that have been approved for the treatment of RA.

DrugBank ID	Name	Summary	Binding Probability	Lowest Docking Score (kcal/mol)
DB00461	Nabumetone	Nabumetone is an NSAID used to treat osteoarthritis and rheumatoid arthritis.	0.89	−7.60
DB00465	Ketorolac	Ketorolac is an NSAID used to treat moderate to severe pain, rheumatoid arthritis, osteoarthritis, ankylosing spondylitis, menstrual disorders, and headaches.	0.81	−7.70
DB00500	Tolmetin	Tolmetin is an NSAID used to treat acute flares of various painful conditions and is used for the long-term management of osteoarthritis, rheumatoid arthritis, and juvenile arthritis.	0.75	−6.40
DB00533	Rofecoxib	Rofecoxib is a COX-2 inhibitor NSAID used to treat osteoarthritis, rheumatoid arthritis, acute pain, primary dysmenorrhea, and migraine attacks.	0.83	−8.10
DB00586	Diclofenac	Diclofenac is an NSAID used to treat the signs and symptoms of osteoarthritis and rheumatoid arthritis.	0.76	−6.40
DB00605	Sulindac	Sulindac is an NSAID used to treat osteoarthritis, rheumatoid arthritis, ankylosing spondylitis, acute subacromial bursitis or supraspinatus tendinitis, and acute gouty arthritis.	0.77	−7.70
DB00712	Flurbiprofen	Flurbiprofen is an NSAID used to treat the signs and symptoms of osteoarthritis and rheumatoid arthritis.	0.70	−7.50
DB00788	Naproxen	Naproxen is an NSAID used to treat rheumatoid arthritis, osteoarthritis, ankylosing spondylitis, polyarticular juvenile idiopathic arthritis, tendinitis, bursitis, acute gout, primary dysmenorrhea, and mild to moderate pain.	0.72	−8.00

**Table 4:** Comprehensive evaluation of SCGFb binding to the candidate molecules with the highest similarities using the DrugBAN model, molecular docking, and molecular dynamics simulations.

Ligand	PubChem CID	Binding Probability	Docking Score (kcal/mol)	Binding Free Energy (kcal/mol) ^1^
3-(4-Hydroxy-phenyl)-4-(4-methanesulfonyl-phenyl)-5H-furan-2-one	10359269	0.80	−7.6	−11.2 ± 2.5
Solaraze	3032	0.85	−5.9	−9.8 ± 2.1
trans-Sulindac	1548885	0.83	−7.2	−11.4 ± 4.8
2-[6-Fluoro-3-(4-methanesulfinyl-benzylidene)-2-methyl-3H-inden-1-yl]-N- hydroxy-N-methyl-acetamide	44305866	0.96	−7.2	−14.4 ± 2.7
Sulindac sulfide	5352624	0.77	−7.6	−16.0 ± 3.9
sodium;(2R)-2-(6-methoxy-2-naphthyl) propionate	6926388	0.86	−6.1	Not stable
2-(6-Methoxynaphthalen-2-yl)-3-methylbutanoic acid	116964097	0.77	−6.7	−8.8 ± 3.5
2-(6-Ethoxynaphthalen-2-yl)propanoic acid	20334995	0.72	−6.2	Not stable

^1^ Calculated by MM-GBSA.

## Data Availability

Data is contained within the article.

## Supplementary Materials

The following supporting information can be downloaded at: https://www.mdpi.com/article/10.3390/ijms26125686/s1.

## Abbreviations

The following abbreviations are used in this manuscript:

AUROC	Area under the receiver operating characteristic
BMI	Body mass index
GWAS	Genome-wide association study
IL6	Interleukin-6
IVW	Inverse variance weight
MD	Molecular dynamics
MIP1b	Macrophage inflammatory protein-1b
MR	Mendelian randomization
MR PRESSO	MR pleiotropy residual sum and outlier
OR	Odds ratio
RA	Rheumatoid arthritis
RANKL	Receptor activator of nuclear factor κB ligand
SCGFb	Stem cell growth factor-beta
SNPs	Single nucleotide polymorphisms
TNF	Tumor necrosis factor
TRAIL	TNF-related apoptosis inducing ligand
WM	Weighted-median
