# Supplementary material for: Identifying Targets and Drugs for Rheumatoid Arthritis Stratified Therapy Using Mendelian Randomization and a Pretraining Model
Source: Int J Mol Sci. 2025 Jun 13;26(12):5686. doi: 10.3390/ijms26125686 (PMC12193122; doi:10.3390/ijms26125686)
Supplement: Supplementary file 1 [file ijms-26-05686-s001.zip › Supplementary figures.pdf]

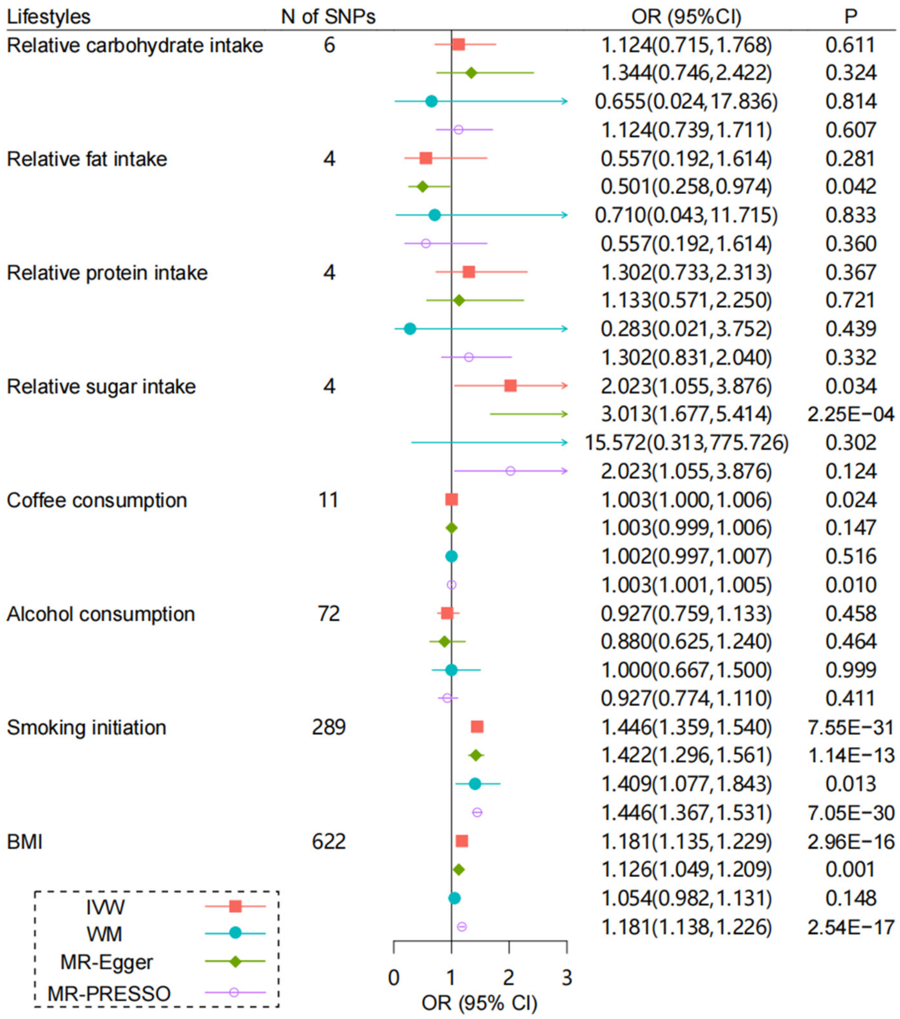

Figure S1. Two-sample MR results for the causal effects of lifestyle factors on RA overall using four different methods.

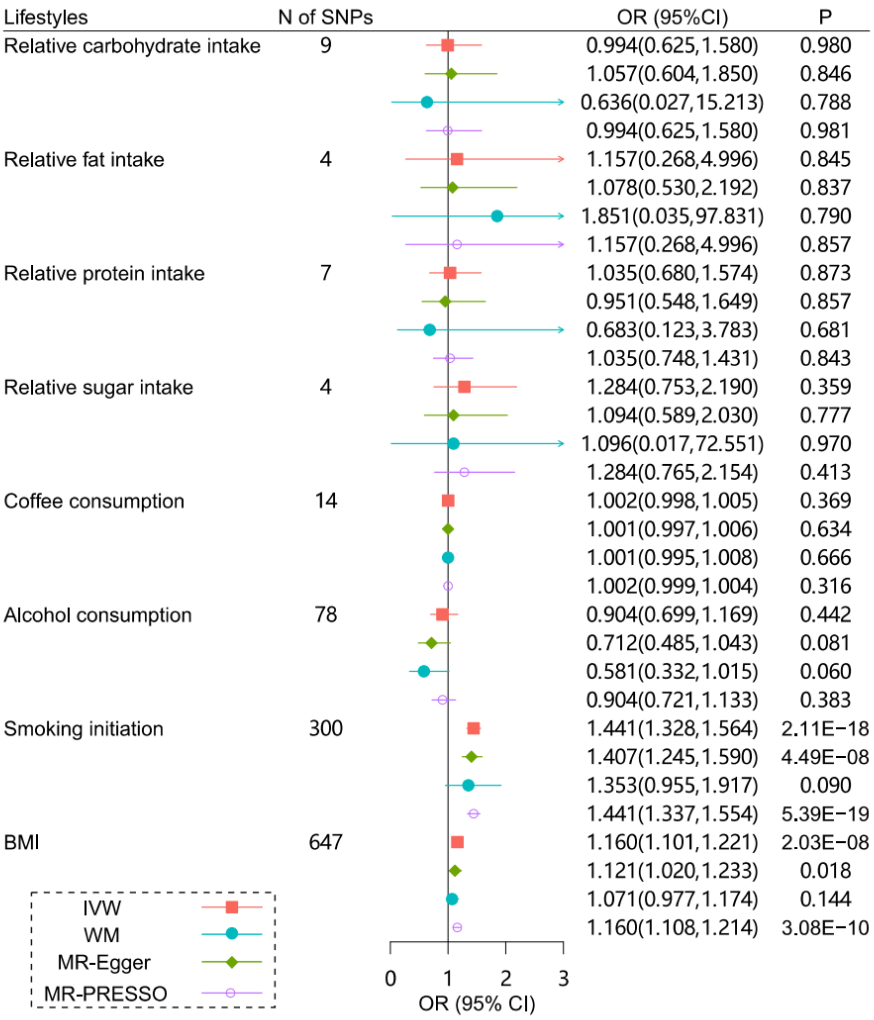

Figure S2. Two-sample MR results for the causal effects of lifestyle factors on seropositive RA using four different methods.

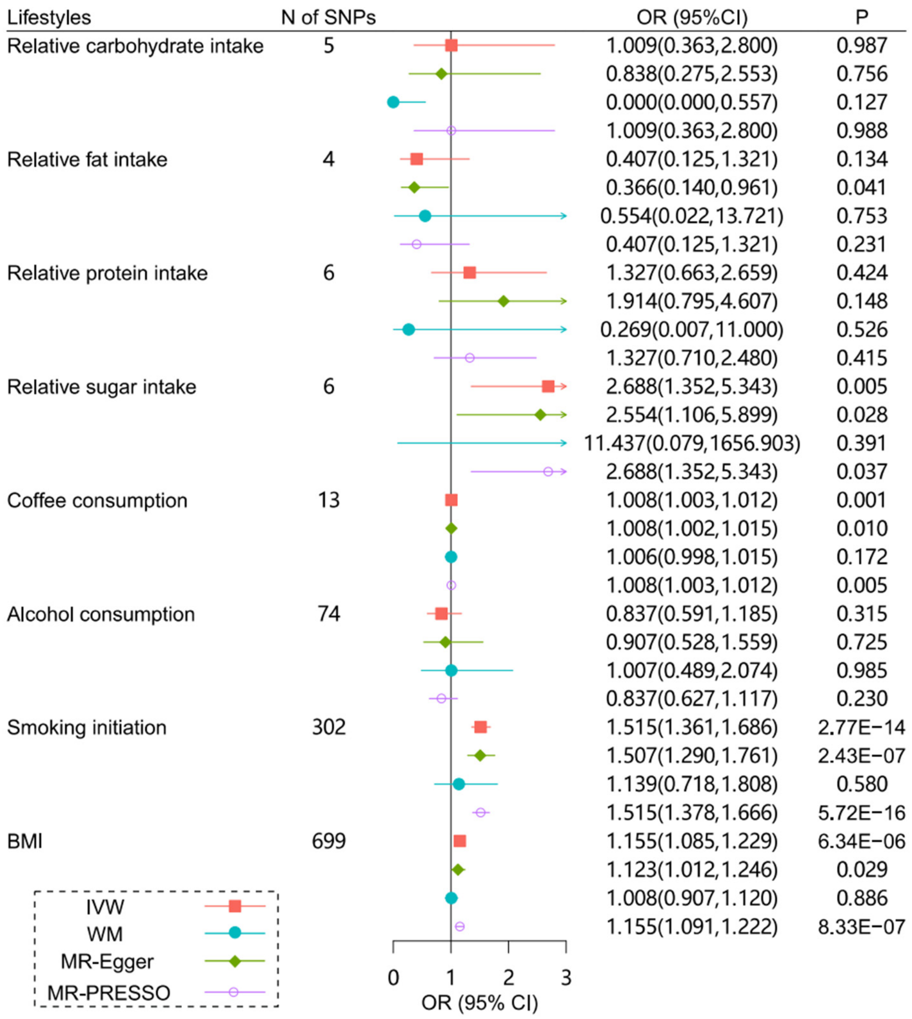

Figure S3. Two-sample MR results for the causal effects of lifestyle factors on seronegative RA using four different methods.

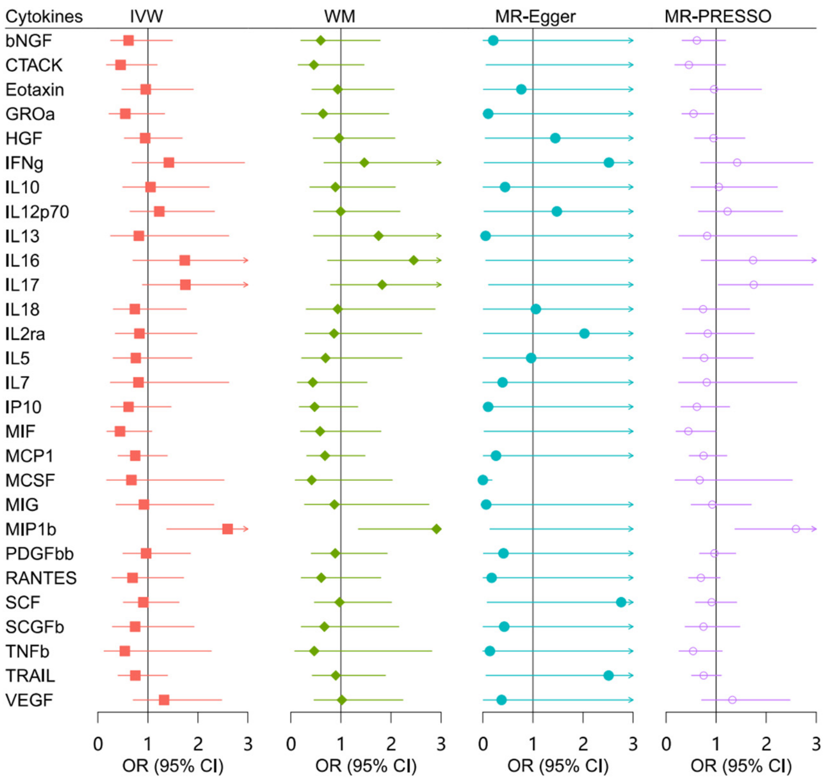

Figure S4. The first-step MR results for the causal effects of relative sugar intake on 28 cytokines using four different methods.

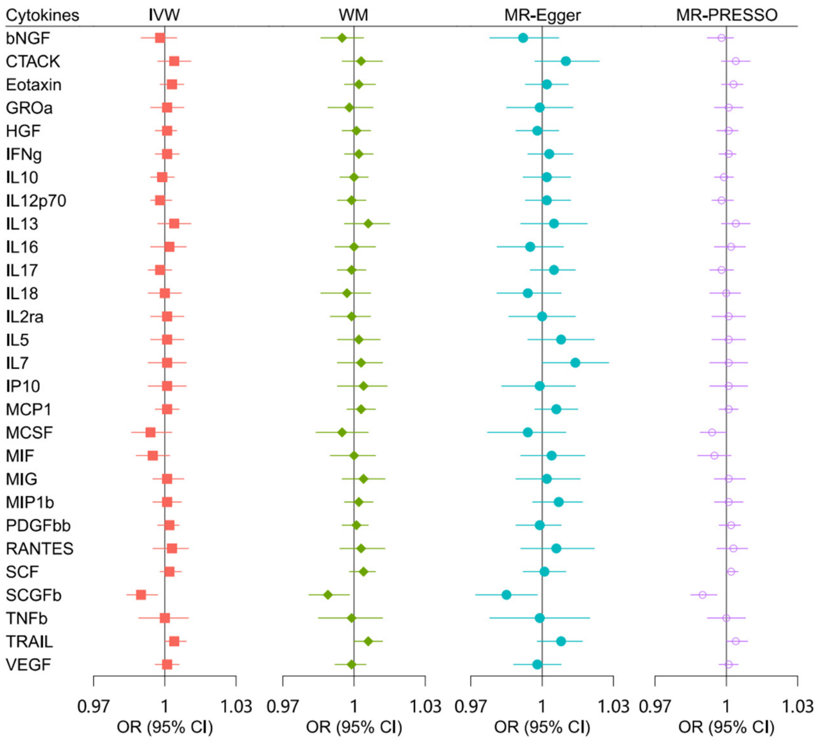

Figure S5. The first-step MR results for the causal effects of coffee consumption on 28 cytokines using four different methods.

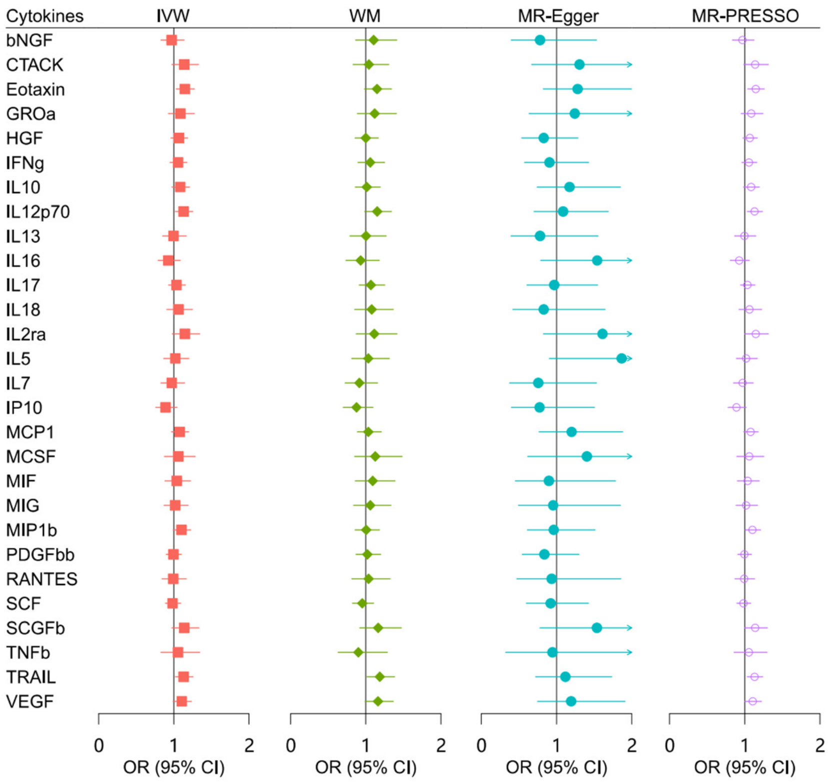

Figure S6. The first-step MR results for the causal effects of smoking initiation on 28 cytokines using four different methods.

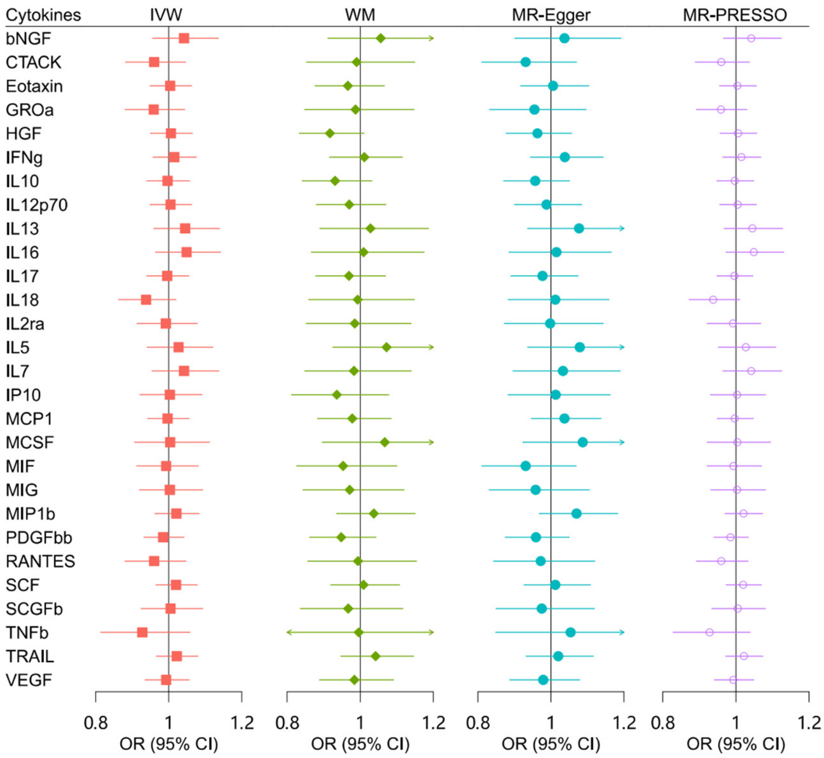

Figure S7. The first-step MR results for the causal effects of BMI on 28 cytokines using four different methods.

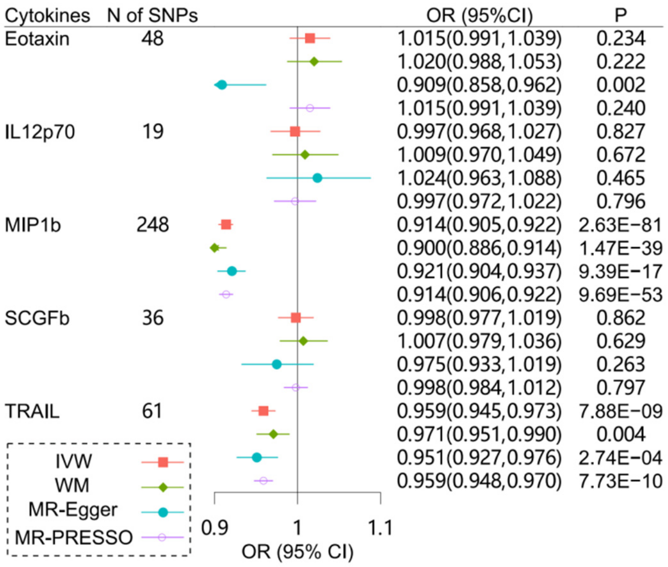

Figure S8. The second-step MR results for the causal effects of cytokines on RA overall using four different methods.

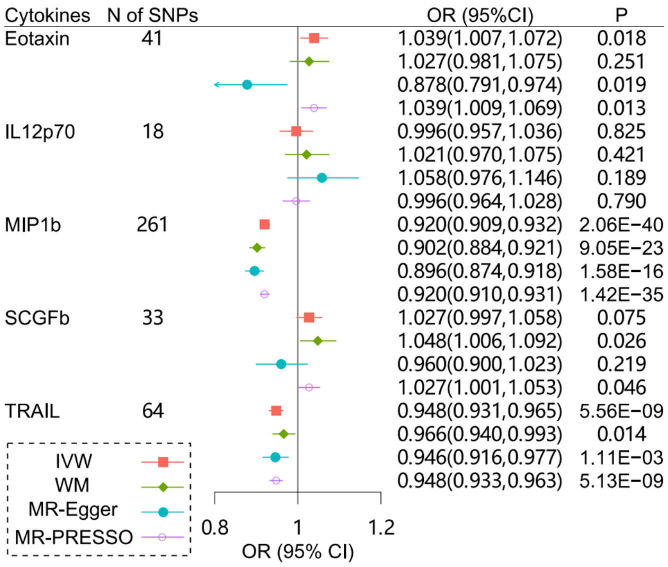

Figure S9. The second-step MR results for the causal effects of cytokines on seropositive RA using four different methods.

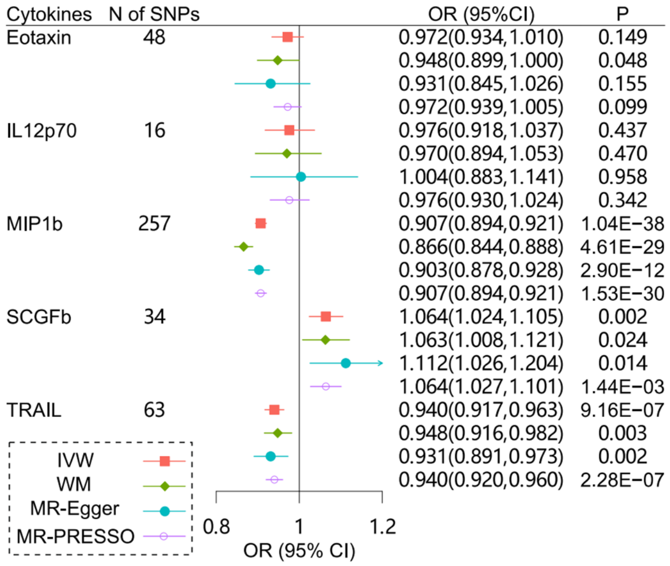

Figure S10. The second-step MR results for the causal effects of cytokines on seronegative RA using four different methods.

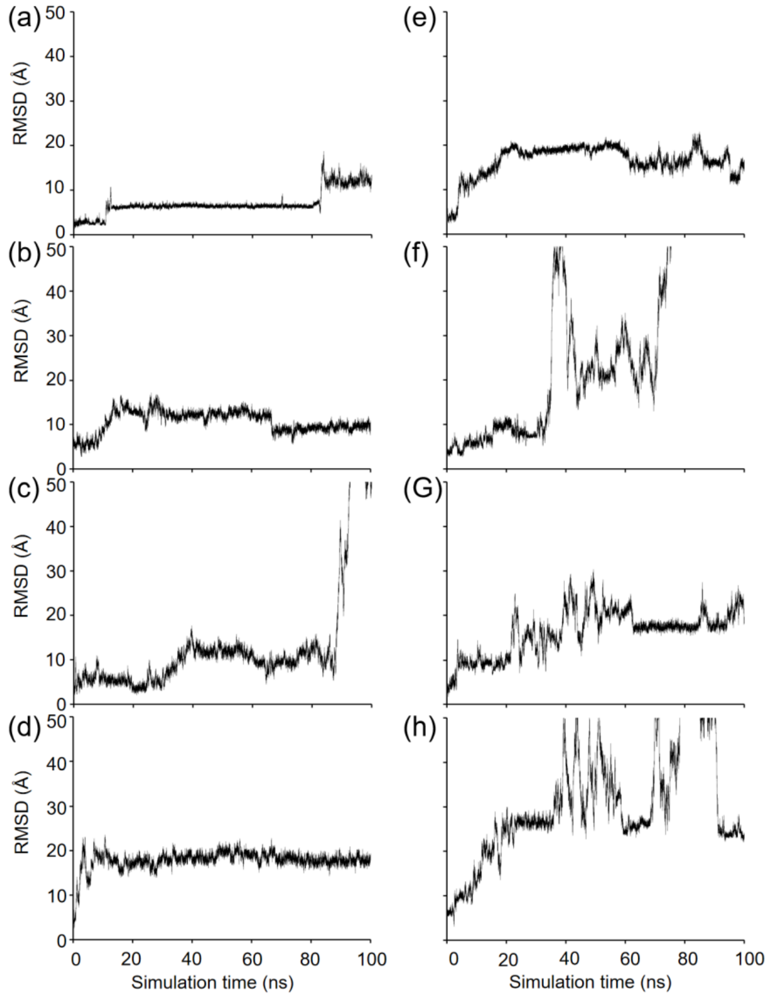

Figure S11. The root mean square deviation (RMSD) for the candidate molecules interacting with SCGFb. PubChem CID: (a) 10359269; (b) 3032; (c) 1548885; (d) 44305866; (e) 5352624; (f) 6926388; (g) 116964097; (h) 20334995.
